# Diversity and Function of Phage Encoded Depolymerases

**DOI:** 10.3389/fmicb.2019.02949

**Published:** 2020-01-10

**Authors:** Leandra E. Knecht, Marjan Veljkovic, Lars Fieseler

**Affiliations:** Institute of Food and Beverage Innovation, Zurich University of Applied Sciences, Wädenswil, Switzerland

**Keywords:** bacteriophage, depolymerase, polysaccharide, capsule, lipopolysaccharide

## Abstract

Bacteriophages of the *Podoviridae* family often exhibit so-called depolymerases as structural components of the virion. These enzymes appear as tail spike proteins (TSPs). After specific binding to capsular polysaccharides (CPS), exopolysaccharides (EPS) or lipopolysaccharide (LPS) of the host bacteria, polysaccharide-repeating units are specifically cleaved. Finally, the phage reaches the last barrier, the cell wall, injects its DNA, and infects the cell. Recently, similar enzymes from bacteriophages of the *Ackermannviridae*, *Myoviridae*, and *Siphoviridae* families were also described. In this mini-review the diversity and function of phage encoded CPS-, EPS-, and LPS-degrading depolymerases is summarized. The function of the enzymes is described in terms of substrate specificity and applications in biotechnology.

## Phage Encoded Depolymerases Cleave Surface Decorating Polysaccharides of Bacteria

Bacteriophages (phages) are viruses, which infect bacteria. The infection begins with the adsorption, the recognition of specific ligands at the host surface by tail fiber or tail spike proteins (TSPs). This adsorption process mostly determines host specificity. In many cases, a bacteriophage infects a particular serotype of the target bacterium only. Due to the high host specificity, the accompanying microflora of a target bacterium remains unharmed during phage attack. This is why bacteriophages and phage-derived enzymes became a promising alternative for detection and control of bacterial pathogens in agriculture, food, and medical applications.

Bacterial cell surface decorating polysaccharides, e.g., capsular polysaccharides (CPS), exopolysaccharides (EPS) or lipopolysaccharide (LPS), exhibit important functions in biofilm production, virulence, and in the interaction with bacteriophages. LPS very often serves as a ligand for phage tail fiber proteins during adsorption to the host cell membrane. CPS and EPS layers can reduce the efficacy of plating of some bacteriophages. In these cases, the capsule sterically inhibits the adsorption of the phages to the primary phage receptor. In mutant strains lacking the protective capsule, adsorption of these phages is significantly increased. Other phages, however, adapted to this challenge. These phages bind and digest CPS and EPS antigens, thereby drilling a tunnel through the capsules. After contact with the next physical barrier, the cell wall, the DNA is injected and the bacterium is successfully infected. Bacteria lacking the capsular antigens cannot be infected by these phages. This indicates that these phages do not only overcome the capsule barrier – they highly depend on it for host recognition and infection. Capsules should therefore be regarded as the primary receptor of such phages. However, the steps between capsule binding and cleaving and finally phage DNA injection into the host cell still remain unclear.

The majority of bacteriophages, which rely on bacterial polysaccharides for infection, belong to the *Podoviridae* family. In addition, an interaction with the bacterial capsule is also evident for some phages belonging to other phage families. Since comparative genomics of bacteriophage genomes enables sophisticated analyses of phylogenetic relationships and revealed a better understanding of bacteriophage evolution, phage taxonomy is currently changing and new phage genera are established. Table 1 provides an overview of the currently used taxonomy in bacteriophages, which interact with bacterial polysaccharides via so-called depolymerases.

**TABLE 1:** Tail spike proteins (TSP) in bacteriophages of different phylogenetic affiliation.

**Phage**	**Bacterial host**	**Family (Subfamily)**	**Genus**	**TSP**	**Accession no.**	**Specificity**	**Remarks**	**References**
**Phage genera encoding one TSP**								
K1F	*E. coli*	*Podoviridae Autographivirinae*	Teseptimavirus (T7virus)	1 TSP EndoNF	Q04830	CPS K1	conserved gp17_T__7_-like N-terminal domain in EndoNF	Stummeyer et al., 2006
L1	*E. amylovora*	*Podoviridae Autographivirinae*	Teseptimavirus (T7virus)	1 TSP DpoL1	YP_007005466	EPS	conserved gp17_T__7_-like N-terminal domain; diversity of amylovoran has not been studied yet	Born et al., 2014
PHB01	*P. multocida*	*Podoviridae Autographivirinae*	Teseptimavirus (T7virus)	1 TSP	ASD51051	D		Chen et al., 2019b
PHB02	*P. multocida*	*Podoviridae Autographivirinae*	Teseptimavirus (T7virus)	1 TSP Dep-ORF8	ARV77571	A		Chen et al., 2018
Petty	*A. baumannii*	*Podoviridae Autographivirinae*	Phikmvvirus	1 TSP Dpo1	YP_009006536	nd		Hernandez-Morales et al., 2018
NTUH-K2044-K1-1	*K. pneumoniae*	*Podoviridae Autographivirinae*	Phikmvvirus	1 TSP ORF34	YP_009098385	CPS K1		Lin et al., 2014
LKA1	*P. aeruginosa*	*Podoviridae Autographivirinae*	Phikmvvirus	1 TSP Gp49	YP_001522890	LPS	Cleaves the LPS B-band	Olszak et al., 2017
P22	*S. enterica*	*Podoviridae*	Lederbergvirus	1 TSP TSP	NP_059644	LPS Typhimurium Enteritidis		Steinbacher et al., 1996
Sf6	*S. flexneri*	*Podoviridae*	Lederbergvirus	1 TSP TSP	NP_958190	LPS Y-serotype		Chua et al., 1999
HK620	*E. coli*	*Podoviridae*	Lederbergvirus	1 TSP TSP	NP_112090	LPS O18A1		Barbirz et al., 2008
Fri1	*A. baumannii*	*Podoviridae Autographivirinae*	Friunasvirus (Fri1virus)	1 TSP	YP_009203055	nd		
AS11	*A. baumannii*	*Podoviridae Autographivirinae*	Friunasvirus (Fri1virus)	1 TSP				Popova et al., 2017
AS12	*A. baumannii*	*Podoviridae Autographivirinae*	Friunasvirus (Fri1virus)	1 TSP				Popova et al., 2017
AB6	*A. baumannii*	*Podoviridae Autographivirinae*	Friunasvirus (Fri1virus)	1 TSP				Lai et al., 2016
IME200	*A. baumannii*	*Podoviridae Autographivirinae*	Friunasvirus (Fri1virus)	1 TSP Dpo48	ALJ97635	nd		Liu et al., 2019
B3	*A. baumannii*	*Podoviridae Autographivirinae*	Friunasvirus (Fri1virus)	1 TSP Gp42	ASN73401	CPS K2	conserved gp17_T__7_-like N-terminal domain	Oliveira et al., 2019b
P1	*A. baumannii*	*Podoviridae Autographivirinae*	Friunasvirus (Fri1virus)	1 TSP Gp43	ASN73504	nd	less specific	Oliveira et al., 2017
P2	*A. baumannii*	*Podoviridae Autographivirinae*	Friunasvirus (Fri1virus)	1 TSP Gp48	ASN73558			Oliveira et al., 2017
P3	*A. baumannii*	*Podoviridae Autographivirinae*	Friunasvirus (Fri1virus)	1 TSP		nd	less specific	Oliveira et al., 2017
N1	*A. baumannii*	*Podoviridae Autographivirinae*	Friunasvirus (Fri1virus)	1 TSP		CPS K2		Oliveira et al., 2017
B1	*A. baumannii*	*Podoviridae Autographivirinae*	Friunasvirus (Fri1virus)	1 TSP Gp45	ASN73353	CPS K9		Oliveira et al., 2017
B2	*A. baumannii*	*Podoviridae Autographivirinae*	Friunasvirus (Fri1virus)	1 TSP				Oliveira et al., 2017
B4	*A. baumannii*	*Podoviridae Autographivirinae*	Friunasvirus (Fri1virus)	1 TSP		nd	less specific	Oliveira et al., 2017
B5	*A. baumannii*	*Podoviridae Autographivirinae*	Friunasvirus (Fri1virus)	1 TSP Gp47	ASN73455	CPS K9		Oliveira et al., 2017
B6	*A. baumannii*	*Podoviridae Autographivirinae*	Friunasvirus (Fri1virus)	1 TSP		nd	less specific	Oliveira et al., 2017
B7	*A. baumannii*	*Podoviridae Autographivirinae*	Friunasvirus (Fri1virus)	1 TSP		nd	less specific	Oliveira et al., 2017
B8	*A. baumannii*	*Podoviridae Autographivirinae*	Friunasvirus (Fri1virus)	1 TSP		CPS K9		Oliveira et al., 2017
IME180	*P. aeruginosa*	*Podoviridae*	nd	1 TSP Gene2	ATG86239	CPS		Mi et al., 2019
KN1-1	*K. pneumoniae*	*Podoviridae*	nd	1 TSP KN1dep	BBF66844	CPS KN1		Pan et al., 2019
KN4-1	*K. pneumoniae*	*Podoviridae*	nd	1 TSP KN4dep		CPS KN4		Pan et al., 2019
JA1	*V. cholera*	*Podoviridae*	nd	1 TSP		LPS O139		Linnerborg et al., 2001
E15	*S. enterica*	*Podoviridae*	nd	1 TSP TSP (gp20)		nd		Guichard et al., 2013
EcoO78	*E. coli*	*Myoviridae*	nd	1 TSP Dpo42		unknown	only distantly related to other depolymerases	Guo et al., 2017
B9	*A. baumannii*	*Myoviridae*	nd	1 TSP Gp69	AWD93192	CPS K45/K30	α-helical protein	Oliveira et al., 2019a
0507-KN2-1	*K. pneumoniae*	*Myoviridae*	Viunavirus (ViIvirus)	1 TSP ORF96	YP_008532047	CPS KN2		Hsu et al., 2013
RAY	*E. amylovora*	*Myoviridae*	nd	1 TSP Gp76	ANH51857	EPS nd	giant jumbophage; Dpo is active on *Pantoea* sp.	Sharma et al., 2019
**Phage genera encoding two TSPs**								
SP6	*S. enterica*	*Podoviridae Autographivirinae*	Zindervirus (SP6virus)	2 TSPs Gp46 Gp47	NP_853609 NP_853610	LPS Typhimurium/Enteritidis Newport/Kentucky		Gebhart et al., 2017
K1-5	*E. coli*	*Podoviridae Autographivirinae*	Zindervirus (SP6virus)	2 TSPs EndoNE (ORF47) K5 lyase (ORF46)	YP_654148 YP_654147	CPS K1 K5	Gp37 serves as an adapter protein that interconnects the 2 Dpo with the capsid	Scholl et al., 2002; Leiman et al., 2007
K1E	*E. coli*	*Podoviridae Autographivirinae*	Zindervirus (SP6virus)	1 TSP EndoNE (ORF47)	YP_425013	CPS K1	The EndoNE neighboring ORF46 is truncated;	Stummeyer et al., 2006; Leiman et al., 2007
K5	*E. coli*	*Podoviridae Autographivirinae*	Zindervirus (SP6virus)	1 TSP KflA		CPS K5		Thompson et al., 2010
K5-2	*K. pneumoniae*	*Podoviridae Autographivirinae*	Przondovirus (KP32virus)	2 TSPs ORF37 ORF38	APZ82804 APZ82805	CPS K5 K30, K69	conserved gp17_T__7_-like N-terminal domain in ORF37, but not in ORF38	Hsieh et al., 2017
K5-4	*K. pneumoniae*	*Podoviridae Autographivirinae*	Przondovirus (KP32virus)	2 TSPs ORF37 ORF38	APZ82847 APZ82848	CPS K5 K8	conserved gp17_T__7_-like N-terminal domain in ORF37, but not in ORF38	Hsieh et al., 2017
KP32	*K. pneumoniae*	*Podoviridae Autographivirinae*	Przondovirus (KP32virus)	2 TSPs KP32gp37 Kp32gp38	YP_003347555 YP_003347556	CPS K3 K21	conserved gp17_T__7_-like N-terminal domain in KP32gp37, but not in KP32gp38	Majkowska-Skrobek et al., 2018
IME321	*K. pneumoniae*	*Podoviridae Autographivirinae*	Przondovirus (KP32virus)	1 TSP Dp42	AXE28435	CPS KN1		Wang et al., 2019
KN3-1	*K. pneumoniae*	*Podoviridae Autographivirinae*	nd	2 TSPs KN3dep KN56dep		CPS KN3 KN56	closely related to phage K5-2	Pan et al., 2019
KpV41	*K. pneumoniae*	*Podoviridae Autographivirinae*	Drulisvirus (KP34virus)	2 TSPs ORF46 ORF55	ALO80736 KT964103	CPS K1 unknown	conserved gp17_T__7_-like N-terminal domain in ORF46, but not in ORF55; no experimental evidence	Solovieva et al., 2018
KpV71	*K. pneumoniae*	*Podoviridae Autographivirinae*	Drulisvirus (KP34virus)	1 TSP Dep_kvp71	AMQ66478	CPS K1		Solovieva et al., 2018
KpV74	*K. pneumoniae*	*Podoviridae Autographivirinae*	Drulisvirus (KP34virus)	1 TSP Dep_kvp74	APZ82768	CPS K2		Solovieva et al., 2018
KLPN1	*K. pneumoniae*	*Siphoviridae Tunaviridae*	Webervirus (T1virus)	2 TSPs ORF34 ORF35	YP_009195374 YP_009195375	CPS K1		Hoyles et al., 2015
KP36	*K. pneumoniae*	*Siphoviridae Tunaviridae*	Webervirus (T1virus)	1 TSP Gp50	YP_009226011	CPS K63		Majkowska-Skrobek et al., 2016
**Phage genera encoding multiple TSPs**								
CBA120	*E. coli S. enterica*	*Ackermannviridae Cvivirinae*	Kuttervirus	4 TSPs TSP1 TSP2 TSP3 TSP4	AEM91896 AEM91897 AEM91898 AEM91899	LPS *S.* Minnesota *E. coli* 0157 *E. coli* O77 *E. coli* O78	Gp10_T__4_-like protein interconnects the four TSPs	Plattner et al., 2019
K64-1	*K. pneumoniae*	*Myoviridae*	Alcyoneusvirus	11 TSPs S1-1 S1-2 S1-3 S2-1 S2-2 S2-3 S2-4 S2-5 S2-6 S2-7 S2-8	BAW85694 BAW85692 BAW85693 BAW85695 BAW85696 BAW85697 BAW85698 BAQ02780 BAW85699 BAW85700 BAW85701	CPS K11 KN4 K21 KN5 K25 K35 K1 K64 K30,K69 nd nd	9 Dpo were functionally characterized	Pan et al., 2017

*Family, Subfamily, and Genus names are provided according to the international committee on the taxonomy of viruses (ICTV). Genus names in brackets refer to previously recommended genus names for ease of understanding. Phage genera are grouped according to the number of TSPs encoded in the phage genomes. The substrate specificity of a single enzyme is provided, if known. nd, not determined.*

A depolymerase is a structural component of the adsorption apparatus, which facilitates binding and digestion of capsules. The name indicates that the repeating unit of a polysaccharide is cleaved and disintegrated. Biochemically, depolymerases are divided into two groups, lyases and hydrolases. Lyases, in contrast to hydrolases, cleave their substrates non-hydrolytically, meaning that no water molecule is released after substrate cleaving. Most of the well-characterized phage encoded depolymerases, which target EPS or LPS O-polysaccharides, are lyases (Tomlinson and Taylor, 1985; Linnerborg et al., 2001; Olszak et al., 2017). They generally feature a great diversity in substrate specificity. However, it could well be that a particular cleavage site is present in different polysaccharide types, thereby allowing the enzyme to act on two different substrates. LPS-targeting enzymes are mostly referred to as TSP, while capsule-targeting enzymes are often termed depolymerases (Dpo). The term depolymerase can refer to any generic protein that is able to degrade polymers. From this perspective, phage encoded endolysins are also depolymerases, since they cleave the peptidoglycan, a bacterial polysaccharide in general in an hydrolase manner (Schmelcher and Loessner, 2016). However, this review will mainly focus on CPS, EPS, and LPS degrading depolymerases.

**FIGURE 1:**
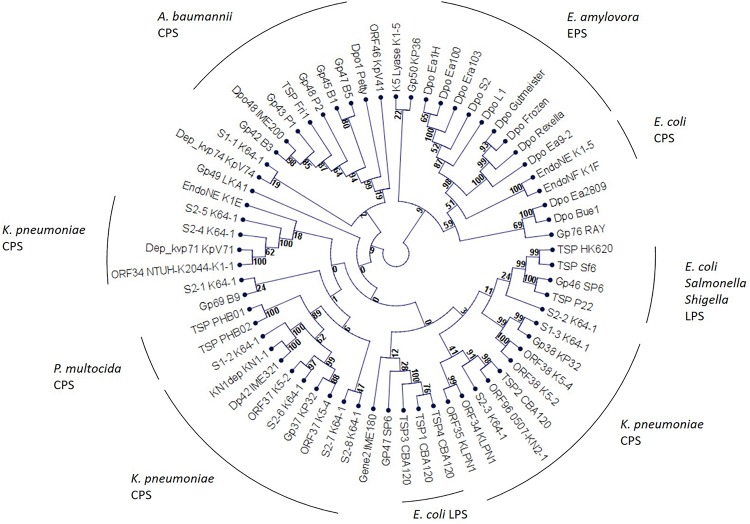
Phylogenetic analysis of phage encoded tail spike proteins. The unrooted cladogram was calculated using neighbor joining and jukes-cantor correction. Bootstrap values are indicated at the nodes. A bootstrap value of 90 is considered significant.

As these enzymes protrude from the virion and form spike-like structures we would therefore, instead of referring to them as depolymerase, use the term TSP in this review. On a structural level these enzymes are highly conserved (Latka et al., 2017). In general, they are homotrimers. A monomer exhibits parallel right-handed β-helices. Due their tertiary structure, TSPs are very stable enzymes. They remain functional at elevated temperatures of up to 80°C and acidic conditions (pH 5.0) and are also protease- and SDS-resistant. From this perspective, TSPs seem to be suitable for several biotechnological applications (Majkowska-Skrobek et al., 2016; Latka et al., 2017; Oliveira et al., 2019a; Wang et al., 2019). A phylogenetic analysis of TSPs partly revealed a substrate-specific clustering, instead of a phage genus-related clustering. This suggests, that TSPs of phages infecting a particular host are more closely related to each other than to other TSPs (Figure 1).

When a phage with an active TSP forms a plaque in soft agar overlays on a capsulated host bacterium, the clear center of the plaque is often surrounded by a turbid zone. This halo formation is regarded a hallmark of TSP activity and can be used to rapidly identify capsule targeting phages. In some cases, the halo even increases during prolonged incubation. Although the TSP strips the bacteria and removes the capsule, it does not necessarily affect bacterial growth *in vitro.* As a hypothesis, halo formation could be due to the TSP activity in diffusing offspring virions, which do not infect the remaining bacteria. Likely bacteria surrounding a plaque exhibit an altered physiological state, rendering them insensitive to infection. As a second hypothesis, a halo could be formed due to soluble, free TSPs. A TSP could be released as a free enzyme, if an alternative start codon is used for translation. As the free TSP will strip the bacteria of their protective capsule, the bacteria will thereby become insensitive toward infection by lyase bearing virions. Hence, the free TSP would inhibit phage adsorption and proliferation. In biofilms, where the bacteria produce large amounts of EPS, high levels of free TSPs could protect the bacteria from phage attack, which would be contra productive for the bacteriophage. On the contrary, free TSPs could be useful in terms of spreading progeny viruses after infection from the host cell and the biofilm, respectively. Future studies should therefore address the ecological function of free TSPs on the spread of phages under natural conditions, e.g., in a multi-species biofilm.

The most extensively studied TSPs are podovirus derived. Hypothetically, podoviruses may rely more on such lyases than phages of the other phage families, because they have short tails and more difficulties in reaching the internal host receptor. In recent years, however, research on similar non-podoviral enzymes is coming to the fore. Apparently, bacteriophages of the *Ackermannviridae*, *Myoviridae*, and *Siphoviridae* family exhibit multiple putative lyases, which await further characterization.

## TSPs Targeting Capsular Polysaccharides (CPS)

A capsule is the outer layer that surrounds a bacterium (Limoli et al., 2015; Wen and Zhang, 2015). It consists of CPS, also termed K-antigens in *Escherichia coli*, *Klebsiella pneumonia*, and *Acinetobacter* spp. or Vi-antigens in *Salmonella* and *Citrobacter freundii*, respectively, which are connected to the outer membrane of Gram-negative bacteria via a lipid anchor (Follador et al., 2016; Hu et al., 2017; Sachdeva et al., 2017; Arbatsky et al., 2018). The alginate of *Pseudomonas aeruginosa* is also referred to as a capsule although it rather represents an EPS (Wen and Zhang, 2015). CPS usually exhibits a size of 100 kDa. Hence, they are much larger than other cell wall associated polysaccharides such as the LPS (Wen and Zhang, 2015). However, some long-chain LPS molecules, like the O111-antigen in *E. coli* or the O19- and O57-antigen in *Proteus vulgaris*, are also considered as capsules. CPS are chemically extremely diverse. They are composed of repeating oligosaccharide units, which can be either linear or branched. Not only monosaccharide constituents, but also glycosidic linkages, and substitutions with non-carbohydrates vary. While *P. aeruginosa* exhibits only one serotype, up to 80 different K-antigens have been described in *E. coli* and *K. pneumoniae* so far (Sachdeva et al., 2017). In *Acinetobacter baumannii* more than 125 capsule synthesis loci were identified (Arbatsky et al., 2018), but information on CPS structures is rather limited and typing schemes are not available yet (Limoli et al., 2015). As CPS can be highly immunogenic, many vaccination strategies rely on CPS-based vaccines. CPS mutants and non-capsulated bacteria are often highly attenuated in virulence. This is why CPS is often regarded as a major virulence factor in pathogenic bacteria (Sachdeva et al., 2017).

## The K1 Capsule of Neuropathogenic *E. coli* is Efficiently Removed by Phage Encoded TSPs

Particular strains of *Escherichia coli* predominately cause bacterial meningitis and septicemia in newborn infants. The majority of these neuropathogenic *E. coli* express the capsular K1-antigen, which features an α-2,8-linked poly-N-acetylneuraminic acid (polysialic acid). As this carbohydrate is also part of the glycocalyx, covering cell membranes in human epithelial cells, the *E. coli* K1-antigen is not recognized by the immune system. This renders infections with *E. coli* K1 very severe. Tomlinson and Taylor identified a hydrolytic TSP with N-acetylneuraminidase (endosialidase) activity in bacteriophage K1E, a podovirus of the *Autographivirinae* subfamily, Zindervirus genus, already in 1985 (Tomlinson and Taylor, 1985). In the following years, a similar gene was described in phage K1F (*Autographivirinae* subfamily, Teseptimavirus genus) (Hallenbeck et al., 1987). Other strains of pathogenic *E. coli* exhibit the capsular K5-antigen. The K5-capsule is composed of 4-linked α-N-acetylglucosamine and β-glucuronic acid (N-acetyl heparosin). Bacteriophage K5 (*Autographivirinae* subfamily, Zindervirus genus), a relative of phage K1E, encodes a TSP, which specifically binds and cleaves the K5-capsule (Thompson et al., 2010). For a better overview, every TSP reviewed in this article is listed in Table 1.

With respect to the tail spike architecture, Teseptima- and Zinderviruses differ remarkably from each other. In phage T7, the “type phage” of the Teseptimavirus genus, Gp17 is the tail fiber protein (Cuervo et al., 2013). The N-terminal domain of this tail fiber, which connects the protein to the baseplate, is highly conserved in different species of Teseptimaviruses. Compared to Gp17_T__7_, TSPs in other Teseptimaviruses exhibit high amino acid identities in this N-terminal domain. The structure of the C-terminal part of Gp17_T__7_ has been studied in detail, and it seems that phage T7 uses Gp17 to adsorb to *E. coli* LPS (Garcia-Doval and van Raaij, 2012; Cuervo et al., 2013). The C-terminus of TSPs features the enzymatically active domain, which cleaves the substrate. Due to the high diversity of different polysaccharide substrates and the high diversity in the primary structure of the C-terminal domain, TSPs exhibit a high substrate specificity. All Teseptimaviruses seem to encode a single type of TSP, which is present in six copies per virion.

**FIGURE 2:**
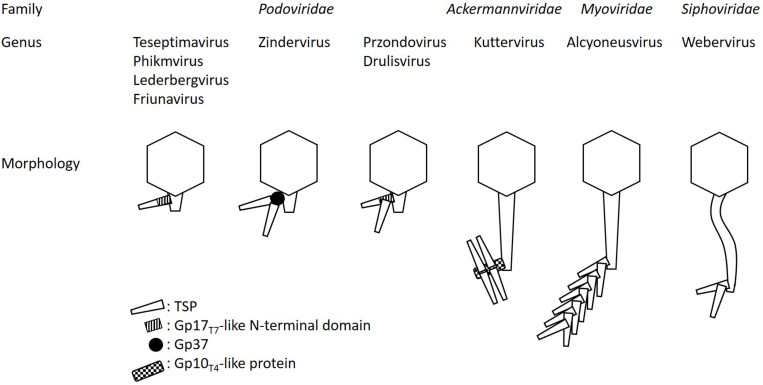
TSP architecture and connection to the virion or baseplate in different phage genera. The schematic is not drawn to scale. For a better overview only one TSP ore branch of TSPs, respectively, is illustrated.

Zinderviruses can encode two TSPs. These enzymes lack the Gp17_T__7_-like N-terminal domain, and are linked to the virion via the small adapter protein Gp37 (Leiman et al., 2007). Figure 2 illustrates the differences in TSP architecture. The adapter can link two different TSPs with different substrate specificities to the virion simultaneously. This enables some members of the Zindervirus genus to infect two different capsular K-antigen types as long as both proteins are active. A prominent example for such a phage is bacteriophage K1-5 (Leiman et al., 2007). This phage encodes both, a K1-specific N-acetylneuraminidase, Gp47, and a K5-specific lyase, Gp46 and can therefore infect K1 and K5 strains of *E. coli*. Each protein is present in six copies per baseplate. Phage K1E also belongs to the Zinderviruses. In contrast to phage K1-5, however, it can only infect K1 strains. CryoEM analyses of K1E revealed that Gp37 serves as an adapter protein, connecting Gp46 and Gp47 to the virion. In agreement with its host range, only the K1-specific N-acetylneuraminidase, Gp47, is enzymatically active, while Gp46 is very small and truncated and does not seem to have an enzymatic activity (Leiman et al., 2007). In phylogenetic analyses, however, the architectural differences in TSPs from Teseptimaviruses, Zinderviruses or other phage genera cannot be reflected (Figure 1).

With respect to phage evolution Stummeyer et al. (2006) argued that K1-specific phages of *E. coli* are not derived from a common ancestor (Stummeyer et al., 2006). It rather seems that each phage is related to a different progenitor type and that the phage gained host specificity through the acquisition of the endosialidase by horizontal gene transfer. New tail spikes could emerge by combining endosialidase domains with the capsid module (gp37) of the respective ancestor.

The K-antigen specific TSPs of coliphages K1E, K1F, K1H, K5, and K30 were therapeutically applied in animal models. Mushtaq et al. (2004) reported that intraperitoneal administration of a recombinant K1-specific endosialidase (20 μg) prevented bacteremia and death from systemic infection in 3-day-old rats. The enzyme did not affect the viability of the K1 *E. coli* strain, but removed the K1 capsule. Thereby, the K1 strain was identified and eliminated by the rat’s complement system (Mushtaq et al., 2004). Lin et al. (2017) found that K1F, K1H, K5, and K30 TSPs could rescue infected mice, but that the K1E enzyme failed to form the expected trimers *in vivo*, which lead to reduced activity (Lin et al., 2017). One year later the same authors tested the robustness of the enzyme across a wider range of conditions. Treatment success rates were reduced by treatment delay. K1- and K5 specific enzymes retained partial efficacy on delay, while K30 lyase did not. Furthermore, the route of administration also affected treatment efficacy (Lin et al., 2018).

In addition to the enzymes derived from podoviruses, Guo et al. (2017) described a novel similar enzyme, Dpo42, derived from the myovirus EcoO78, which infected *E. coli* (Guo et al., 2017). Plaques of this phage exhibit a turbid halo surrounding the clear center. Dpo42 seems to effectively prevent biofilm formation of the tested *E. coli* strains, but could not entirely remove an established biofilm. The structure of the CPS in this particular strain of *E. coli* was not mentioned (Guo et al., 2017). Any structural detail of tail fiber proteins in this phage are also unknown so far. It seems that Dpo42 is only distantly related to other depolymerases.

## Hypermucoviscous Strains of *Klebsiella pneumoniae* Are Sensitive to Serum Killing After TSP Treatment

*Klebsiella pneumoniae*, another member of the *Enterobacteriaceae*, causes nosocomial infections, including pneumonia, urinary tract infections, and to an increasing extent pyogenic liver abscess in humans. The most relevant strains exhibit the K1-, K2-, K5-, K20-, K54-, or K57-antigen (Yu et al., 2008; Hsu et al., 2016). Some experimental data indicate that N-acetylneuraminic acid (polysialic acid) is present in the K1 capsule of *K. pneumoniae*. However, the biochemical composition of many other *K. pneumoniae* capsule antigens, is incomplete and direct links between structural, biochemical, and genetic data for some capsular types are still lacking. In addition, novel K-types, such as capsular antigen types KN1, KN2, amongst others, have just recently been discovered (Yu et al., 2008; Pan et al., 2019). In many strains of *K. pneumoniae* capsule biosynthesis genes have been sequenced. The genomic diversity seems to reflect biochemical diversity of capsular antigens. Notably, genotyping seems to replace traditional biochemical capsule analyses in *Klebsiella* research today.

Bacteriophages targeting various K-antigens of *K. pneumoniae* have been described. Lin et al. (2014) isolated bacteriophage NTUH-K2044-K1-1 which specifically infects capsular type K1. This phage belongs to the *Autographivirinae* subfamily, genus Phikmvvirus, and encodes a single putative lyase (Lin et al., 2014). Solovieva et al. (2018) isolated and characterized eight bacteriophages specific for capsular types K1, K2 or K57, respectively. Two of the eight phages were analyzed in detail and the corresponding TSPs were cloned and functionally characterized. Phage KpV71 and phage KpV74 belong to the *Autographivirinae* subfamily, genus Drulisvirus (Kp34virus). KpV71 infects capsular type K1 and KpV74 capsular type K2. Both phages encoded a single functional enzyme. However, in the KpV71 encoded protein the authors identified two iterative lyase motifs (Solovieva et al., 2018). Any further information on the functionality of the single motifs is not available yet.

Another bacteriophage, IME321, again a member of the *Autographivirinae* subfamily, but from the Przondovirus genus (KP32virus), encodes a functionally active lyase (Dp42) that is specific for capsular type KN1. This enzyme is stable and fully active at a pH range from 5.0 to 12.0. Below pH 5.0 the enzymatic activity decreases significantly. It is also moderately thermo-stable, but if temperature exceeds 55°C the enzyme is inactivated (Wang et al., 2019). Pan et al. (2019) reported on the discovery of KN1-, and KN4-specific phages. Phage KN1-1 and KN4-1 have been allocated to the *Podoviridae* family (Pan et al., 2019). A more detailed phylogenetic analysis of the two viruses has not been performed. Both exhibit a single enzyme.

Hsieh et al. (2017) isolated two bacteriophages, K5-2 and K5-4 of the Przondovirus genus (KP32virus), which encode two capsule-specific TSPs. Shortly thereafter, similar Przondoviruses, phages KP32 and KN3-1 were described to also encode two different TSPs (Majkowska-Skrobek et al., 2018; Pan et al., 2019). K5-4 infects K5 and K8 capsular types, while KP32 adsorbs to K3 and K21 (Hsieh et al., 2017; Majkowska-Skrobek et al., 2018). Phage KN3-1, a podovirus that was not yet allocated to a phage genus, exhibits specificity to capsular types KN3 and KN56, respectively (Pan et al., 2019). Phage K5-2, however, infects three capsular types, e.g., K5, K30, and K69 although it encodes only two TSPs. Hsieh et al. point out that the K30 and K69 antigens differ only in the location of a pyruvic acetal, which seems to be unimportant for adsorption of K5-2 (Hsieh et al., 2017). In all of these phages, the open reading frames ORF37 and ORF38 encode the enzymes (Hsieh et al., 2017; Majkowska-Skrobek et al., 2018).

Solovieva et al. (2018) identified bacteriophage KpV41, a capsular type K1-specific Drulisvirus, which also encoded two different putative lyases (ORF46 and ORF56). Unfortunately, the proteins were not functionally characterized.

Interestingly, Przondo- and Drulisviruses, which feature two separate TSPs per virion, seem to have in common that the upstream encoded enzyme, e.g., ORF37 in phages K5-2 and K5-4, and ORF46 in phage KpV41, exhibits the conserved Gp17_T__7_-like N-terminal domain, which connects the proteins to the baseplate (Hsieh et al., 2017; Solovieva et al., 2018). However, this domain is apparently not present in the downstream encoded TSP, e.g., ORF38 in phages K5-2 and K5-4, and ORF55 in phage KpV41 (Hsieh et al., 2017; Solovieva et al., 2018). Thus, it remains unclear how the two enzymes are connected to the virion in Przondo- and Drulisviruses (Figure 2).

In addition to the podoviruses, several other bacteriophages of the *Myoviridae* and *Siphoviridae* families have been described infecting *K. pneumoniae*. Phage KP36, a member of the *Siphoviridae* family, genus Webervirus (T1virus), specifically infects K63 strains of *K. pneumoniae*. In this phage the active enzyme is encoded by gp50. It retains activity over a pH range from 4.0 to 7.0, but it is not temperature resistant, since the enzymatic activity is abolished at temperatures exceeding 45°C. Interestingly, the authors mentioned, that the *K. pneumoniae* capsular type K63 is structurally identical to the K42 CPS of *E.* coli. Hence, Gp50 is likely to be also active on certain *E. coli* (Majkowska-Skrobek et al., 2016). Phage KLPN1 is another KP36-like siphovirus, genus Webervirus. The phage produced plaques with expanding halos on type K2 capsular strains. Electron microscopy revealed that the tail fibers of this phage appear as elongated spherical structures. The baseplate resembles a rosette-like form with three “leaves.” Based on amino acid sequence similarities ORF34 and ORF35 encode an endo-N-acetylneuraminidase/endosialidase domain. Experimental evidence on the function of these proteins is not available yet. Although the phage KLPN1 specifically infected K2 strains, it may well be that other capsular types are also infected by this phage due to the presence of two active enzymes (Hoyles et al., 2015).

The myovirus 0507-KN2-1 encodes a putative lyase (ORF96) that specifically cleaves the KN2 capsular type (Hsu et al., 2013). Based on genome sequence comparisons, this phage can be allocated to the Viunavirus genus. It forms small clear plaques with a large turbid halo. Probably the most curious myovirus that has been described to specifically interact with capsular antigens in *K. pneumoniae* is bacteriophage K64-1. K64-1 is a member of the *Myoviridae* family, Alcyoneusvirus genus that is currently not allocated to a particular subfamily. K64-1 infects ten different capsular types of *K. pneumoniae*. It encodes 11 different capsule degrading enzymes from which nine have been functionally characterized. Tail fiber structures in this phage are unique, as they appear as branching structures. It is likely that all encoded enzymes are present in the complex baseplate and tail fiber apparatus. The fine structure of these tail fibers is unknown so far (Pan et al., 2017).

The phage-encoded TSPs, which specifically cleave capsules of *K. pneumoniae*, have been applied in various set-ups to specifically control infections caused by the bacteria. In general, it seems that they retain their activity also *in vivo*. Decapsulation of *K. pneumoniae* rendered the bacteria sensitive to serum mediated killing, thereby significantly increasing survival of the infected rodents or of *Galleria mellonella* larvae, respectively (Lin et al., 2014; Majkowska-Skrobek et al., 2016; Majkowska-Skrobek et al., 2018; Pan et al., 2019; Wang et al., 2019). Furthermore, lyases do not seem to disturb other drugs, e.g., antibiotics, used to treat the infection (Majkowska-Skrobek et al., 2016).

Due to the high specificity of a TSP, pathogenic strains of *K. pneumonia* need to be isolated and typed in the clinic, before enzyme therapy can be applied. Mixed infections with strains exhibiting different capsular types are likely more difficult to treat, as long as the two different strains are not differentiated. If the enzyme is inactive on a particular strain, the bacteria cannot be controlled by the immune system. Therefore, rapid and cost-efficient typing methods are essential in order to choose the correct enzyme from a library.

As accurate typing of capsular antigens, either biochemically or genotypically, can be time consuming and laborious, “TSP-typing” would be specific, accurate, and easy. It seems that the purified enzymes even exhibit a greater specificity toward the capsular type than the native virion itself, rendering this technique advantageous compared to classical phage typing.

## Decapsulated *Acinetobacter baumannii* Are Attenuated *In Vivo*

Health care acquired infections by bacteria of the *A. calcoaceticus – A. baumannii* (ACB) complex are increasing worldwide in hospital settings. The bacteria belong to the *Pseudomonadales* order and are therefore closely related to *Pseudomonas* spp. The high number of multi drug resistant strains and the capability of the bacteria to colonize catheters and other medical equipment, hinders effective control and prevention in the clinic. In *A. baumannii* 125 different capsular antigen types have been described (Arbatsky et al., 2018). However, only a few K-types, e.g., K1 and K45, have been identified as virulence factors so far. Like in *K. pneumonia*, the diversity of K-antigen structures has not yet been studied biochemically, but genomically. A typing scheme for the ACB complex is not available yet, but strains of *A. baumannii* have been grouped into K-types. Some of these K-types were allocated to particular sequence types (ST).

Together with several highly similar viruses bacteriophage Fri1 represents an additional phage genus, Friunavirus, of the *Autographivirinae* subfamily. Friunaviruses encode a single TSP per virion. The enzymes again exhibit the N-terminal Gp17_T__7_-like domain for connection with the virion. In the two phages AS11 and AS12 the putative lyase is encoded by ORF 42 and ORF 45, respectively (Popova et al., 2017). From phage AB6 the purified enzyme (ORF 40) was functionally active on the tested phage host strain (Lai et al., 2016). However, a K-type specificity of AS11, AS12, and AB6 has not been reported yet.

Oliveira et al. (2017) isolated and characterized 12 phages, which infected species of the ACB complex. The isolated podoviruses exhibited high synteny in their genomes, although they have been isolated from distantly separated geographical regions, which spanned from America to Europe and Asia. The C-terminal pectate lyase domain of the TSP was often the only difference found among the viral genomes. All phage isolates belong to the *Autographivirinae*, Friunavirus genus. From phage P1, the authors characterized the function of the lyase. The enzyme exhibits the N-terminal gp17_T__7_-like domain. The C-terminal pectate lyase domain was cloned without the N-terminal domain and the authors proved its activity *in vitro*. In spotting tests it was functionally active even at concentrations of 0.1 μg/ml, and it retained its activity even after 2 years of storage at 4°C. As biochemical data on K-types are not available in *A. baumannii*, the authors linked the host range, with the genotype of the respective strains. Bacteriophages B3 and N1 specifically infected K2 strains of *A. baumannii*, while phages B1, B5, and B8 infected K9 strains. Phage B2 targeted a K44 strain. Interestingly, bacteriophages B4, B6, B7, P1 and P3 seem to be less specific, since they infected strains of different K-types. The authors, however, did not mention which strains and if two or more different K-types are infected by these phages. Based on the homologies of the capsular synthesis genes identified in the tested strains to glycosyltransferase sequences in the public databases, the authors hypothesized that the lyase of phages B3 and N1 may rely on pseudoaminic acid and glucopyranose residues in the capsule. The lyases of B1, B5, and B8 may bind to GalNAcA, while phages B4, B6, B7, P1 and P3 may have a preference for GlcNAc structures (Oliveira et al., 2017).

Another member of the *Podoviridae* family, phage Petty, was reported to infect both, strains of *A. baumannii* and *A. nosocomialis* (in total 4/40 strains tested). Unfortunately, the K-types of the respective strains were not mentioned in this study. Petty belongs to the genus Phikmvvirus. The TSP is encoded by gp39 and the protein was named Dpo1. Dpo1 also exhibits the N-terminal gp17_T__7_-like domain. The authors found that the enzymatic activity is enhanced by divalent cations (Hernandez-Morales et al., 2018). Liu et al. (2019) identified the TSP Dpo48 in phage IME200. The enzyme tolerates pH 5 – 9 and temperatures of 70°C. The K-type specificity of Dpo48 is still unknown (Liu et al., 2019). Recently, Oliveira et al. (2019b) characterized gp38 of bacteriophage B3, a member of the *Autographivirinae*. They found that it exhibits the N-terminal gp17_T__7_-like domain and that it is specific for K2-types. The enzyme is thermostable at 60°C (Oliveira et al., 2019b).

A K45/K30-specific TSP (gp69) was identified in bacteriophage B9, which belongs to the *Myoviridae* family (Oliveira et al., 2019a). Interestingly, based on bioinformatics and structure prediction this enzyme is composed of α-helices, and not of β-helices as usual. It remained stable at 80°C and retained its activity at a pH range from 5.0 to 9.0. The authors also compared the development of insensitive bacteria after prolonged incubation with the enzyme and the phage B9, respectively. They found that if bacteria were subjected to the enzyme alone and then re-isolated after incubation, they retained sensitivity. In contrast, bacteria subjected to the bacteriophage developed phage resistance. As a TSP does not kill the bacteria, but remove the capsule only, no selective pressure is applied on the bacteria *in vitro*. From this perspective, the development of resistance to TSPs might be underestimated.

The described TSPs were characterized not only *in vitro*, but also in various animal models *in vivo*. The interaction of the purified enzyme and the target bacteria was analyzed during infection. It seems that every tested enzyme renders the ACB bacteria sensitive to serum killing by the complement system and that infected animals either survive significantly longer, or can be even cured after infection in laboratory settings (Oliveira et al., 2017; Liu et al., 2019; Oliveira et al., 2019a, b). One TSP, Dpo1 of phage Petty, was also tested for its ability to degrade biofilms produced by *A. baumannii*. Although the enzyme removed some biofilm in a few tested strains, the removal amounted to only 20%. The authors concluded that although Dpo1 was active on the capsules of the tested strains, simple degradation of CPS may not be sufficient for removal of adhered *A. baumannii* cells. It is likely that Dpo1 is active against only one particular polysaccharide and *A. baumannii* may produce additional EPS polysaccharides or rely on other factors for biofilm formation (Hernandez-Morales et al., 2018). In addition to these experiments, the high specificity of the enzymes was once more shown to be very useful to establish a fast and reliable typing scheme for ACB complex bacteria.

## TSP Therapy Rescued Mice Infected by *Pasteurella multocida*

*Pasteurella multocida* is part of the oral microflora in many domesticated and agricultural animals. Capsular type A strains usually cause bovine hemorrhagic septicemia and avian cholera. In addition, the bacteria can cause oral diseases. Chen et al. (2018) expressed Dep-ORF8 of bacteriophage PHB02 (Teseptimavirus) recombinantly in *E. coli* and proved that the enzyme is capsular type A specific (Chen et al., 2018). The enzyme was also tested for its efficacy to control *P. multocida in vitro* and *in vivo*. If applied alone, it did not exhibit any detrimental effect on growth of the bacteria *in vitro*. In combination with serum, viable cell counts of *P. multocida* were reduced by 3.5–4.5 logs. Application of the enzyme *in vivo* did not reveal toxic effects. From liver, spleen, kidneys, and lungs of Dep-ORF8 treated mice, an increase of eosinophils, basophiles or other pathological changes were not evident. If mice were infected with the pathogen and later treated with the enzyme, survival of the animals was significantly increased compared to the untreated control group (Chen et al., 2018). A closely related phage, PHB01 (Teseptimavirus), was reported to be capsular type D specific (Chen et al., 2019b). However, PHB01 could not infect every type D strain tested. The authors speculate that these strains may be phage resistant. The TSP of PHB01 was not characterized further yet.

## Two Different Depolymerases Degrade the Capsule in *Bacillus subtilis*

In Gram-positive bacteria, CPS can also be chemically diverse. *Streptococcus pneumoniae* exhibits 98 different capsular serotypes (Paton and Trappetti, 2019). In *Staphylococcus aureus* 11 different capsular serotypes have been described (O’Riordan and Lee, 2004). The capsule contains mainly poly-N-acetylglucosamine. With respect to its function during biofilm formation, it is also termed polysaccharide intercellular adhesion (PIA) (Rohde et al., 2010; Wen and Zhang, 2015). *Bacillus anthracis*, *B. subtilis* or *S. pyogenes* also produce a capsule. However, these capsules belong to just one serotype. *S. pyogenes* produces a hyaluronic acid capsule, which is composed of glucuronic acid and *N*-acetylglucosamine repeating units. *Bacillus* spp. exhibits a rather unusual capsular polypeptide of γ-linked glutamate, e.g., poly-γ-glutamate (γ-PGA). Bacteriophage φNIT1, a member of the *Herelleviridae* family, Nitunavirus genus encodes a γ-PGA hydrolase (PghP) (Kimura and Itoh, 2003). The enzyme hydrolyses γ-PGA to oligopeptides, which are then converted to tri-, tetra-, and penta-γ-glutamates. Interestingly, PghP does not seem to be a structural protein of the φNIT1 virion. Instead, the enzyme is expressed at the end of the phages’ infection cycle and is burst-released from the infected cells. It seems that *pghP* was acquired from a host organism. The structure of PghP was not determined yet. In addition to PghP, a second polysaccharide-degrading enzyme, an endo-levanase (LevP), was identified in φNIT1. LevP cleaves levan, an exopolysaccharide of *B. subtilis*. Again, it seems that also *levP* was acquired from a host organism. LevP is not a TSP, because similar levanases rather exhibit an N-terminal beta- propeller and a C-terminal beta-sandwich (Ernits et al., 2019). Apparently, PghP- and LevP-like proteins are also encoded in many other bacteriophages infecting *B. subtilis* (Ozaki et al., 2017).

## Extracellular Polysaccharide (EPS) Degrading TSPs

Extracellular polysaccharides (EPS) also add to the bacterial surface decoration (Limoli et al., 2015). Unlike CPS, EPS are not connected to the cell wall *per se*, but are secreted from the bacteria (Sachdeva et al., 2017). In general, EPS is considered as one of the major constituents in bacterial biofilms, where they mediate attachment and micro colony formation. Examples for bacterial EPS are alginate, Pel and Psl from *P. aeruginosa*, levan, colonic acid, bacterial cellulose from many phylogenetically different bacterial species, amylovoran from the plant pathogen *Erwinia amylovora*, *Vibrio* polysaccharides, or *B. subtilis* polysaccharides (Limoli et al., 2015). It should be noted that bacteria do not necessarily produce just one particular EPS, but rather a combination of different EPS at the same time. The ability to produce a biofilm, e.g., three-dimensional multilayer structures of bacteria embedded in an EPS matrix, seems to represent the most prominent form of microbial growth in nature. In a biofilm, bacteria are protected from desiccation, UV radiation, limited availability of nutrients, and antibiotics. Bacterial growth in a biofilm features the development of persister cells, which are resistant to environmental stresses and very difficult to eliminate. Absence of EPS decreases the ability of the bacteria to adhere to surfaces or tissues, affects colonization and therefore virulence in pathogenic bacteria.

## In the Fire Blight Pathogen *Erwinia amylovora*, TSPs Enhance Efficacy of Capsule Independent Phages

*Erwinia amylovora* is the causative agent of fire blight, a severe disease of *Rosaceae* plants. To prevent infection, streptomycin is usually applied during the flowering period. However, in some countries, use of streptomycin was banned recently. *E. amylovora* usually infects host blossoms via the stigma and invades the ovary. Later it spreads through the xylem vessels of an infected plant. In the xylem vessels *E. amylovora* produces high amounts of EPS, which leads to ooze formation, necrosis, and canker development. In *E. amylovora* the capsule is mainly composed of amylovoran and levan. Bacteriophage L1, a member of the *Autographivirinae*, genus Teseptimavirus (T7virus), exhibits Dpo_L__1_. The enzyme specifically cleaves amylovoran at the galactose backbone (Born et al., 2014). Any variation in the chemical structure of amylovoran has not been described yet and the majority of tested strains was sensitive to Dpo_L__1_. The enzyme was functionally characterized. It remained active at a pH range of 5.0–7.0 and featured a temperature optimum at 50°C (Born et al., 2014). The Dpo_L__1_ encoding gene was later inserted into the genome of the capsule independent phage Y2, a myovirus. Infection of capsulated wild type bacteria by the recombinant phage Y2:*dpo*_L__1_ revealed an improved efficacy and a more profound reduction of target bacteria compared to the parental phage (Born et al., 2017). Moreover, additional lyase encoding genes were identified in several other bacteriophages infecting *E. amylovora* such as the Zindervirus S2 (Knecht et al., 2018), many N4-like viruses (Thompson et al., 2019), Viunavirus Bue1 (Knecht et al., 2018), and in a giant jumbovirus RAY (Sharma et al., 2019). So far, only the enzymes of Bue1 and Gp76 of phage RAY have been preliminary characterized. The Bue1 enzyme did not exhibit amylovoran degrading activity (Knecht et al. unpublished results). The GP76 enzyme of phage RAY exhibited capsule degrading activity on closely related strains of *Pantoea*, but not on the tested *Erwinia* host strains (Sharma et al., 2019). Future research will help to understand the function of these enzymes.

## Biofilms of *Pseudomonas aeruginosa* Are Destabilized by Alginate-Specific TSPs

*Pseudomonas aeruginosa* is an opportunistic pathogen, which causes major problems in intensive care units. In the environment, the bacteria are typically associated with water and humid habitats. In many countries worldwide absence of *P. aeruginosa* is a microbiological criterion for the safety of drinking water. Depending on the national legislation, the bacteria must not be present in 100 or 300 ml samples, respectively. *P. aeruginosa* can cause severe wound infections and is typically associated with patients suffering from cystic fibrosis (CF). CF patients are prone to infection by mucoid strains, which are difficult to treat by antibiotics. The development of multiple drug resistance (MDR) in *P. aeruginosa* is another factor, which complicates therapy of infected patients. In addition, *P. aeruginosa* is a well known biofilm producer. Biofilms produced by the bacteria are typically thick, as *P. aeruginosa* can produce large amounts of alginate EPS (D-Mannuronate-L-Guluronate). When growing in the biofilm mode, *P. aeruginosa* can exhibit a more than 100-fold greater resistance toward antibiotics. Hence, as long as the bacteria do not develop MDR, TSP aided removal of the alginate EPS could render the bacteria more sensitive to antibiotics. To overcome MDR an increasing number of researchers aims at applying bacteriophages to control the pathogen. However, many *P. aeruginosa*-specific bacteriophages exhibit rather narrow host ranges and a selection of many different bacteriophages applied as a phage cocktail is needed for efficient control.

Glonti et al. (2010) described phage PT-6 (*Podoviridae*), that was biochemically characterized to lyse the alginate capsule of *P. aeruginosa in vitro* (Glonti et al., 2010). However, the genome sequence of phage PT-6 was not determined and a phylogenetic characterization has not been performed so far. Bacteriophage IME180 encodes a TSP in gene 2. Application of the enzyme in serum killing experiments enhanced inactivation of the treated bacteria. In addition, biofilms produced by *P. aeruginosa* could be successfully destroyed, but not completely eliminated by the enzyme (Mi et al., 2019).

Extracellular polysaccharides producing strains of *Pseudomonas putida* have also been controlled by bacteriophages and phage derived lyases. Cornelissen et al. (2011, 2012) identified a putative lyase in bacteriophages φ15 and AF, two members of the *Autographivirinae* subfamily. The authors applied φ15 to degrade biofilms of *P. putida*. As the phage mediated control of the biofilms was less profound than expected, the authors concluded that biofilm formation may even be a mechanism of phage resistance. The lyases were preliminarily studied, but activity on EPS was evident (Cornelissen et al., 2011; Cornelissen et al., 2012).

Judging from these studies it seems that removal of bacterial biofilms by application of lyases remains challenging and that more research is needed to better understand the limits. In natural habitats biofilms are likely produced by phylogenetically distinct bacteria. Hence, the EPS of a natural biofilm may be much more diverse than between different strains of a particular species. Given the high specificity of a lyase, a natural biofilm may not be affected by the action of a single enzyme. However, during infection of plants, animals and humans a particular strain of the pathogen dominates the intrinsic microflora. Hence, a single enzyme is very affective and can be applied as treatment. With the rise of multiple drug resistances in many different pathogenic bacteria and predominantly in the Gram-negatives, lyases represent a very promising treatment option.

## Lipopolysaccharide Degrading TSPs

Lipopolysaccharide as another prominent polysaccharide has a high structural diversity, which defines many different serotypes in Gram-negative bacteria. The LPS is anchored in the bacterial outer membrane through the innermost part the lipid A, a phosphorylated diglucosamine. This lipid A is an endotoxin that causes inflammation in mammals after breakdown of the bacterial cell wall. In its core LPS contains the inner and outer core carbohydrates, e.g., KDO and heptoses, respectively. The core antigens are usually conserved in a particular bacterial species. The protruding O-antigen is composed of repeating units of 3–8 carbohydrates directly linked to the core oligosaccharide. It exhibits a great diversity, giving rise to 160 different O-antigens in *E. coli*, and more than 2500 different serotypes in *Salmonella*. Full length O-chains render a colony smooth, whereas truncated LPS molecules render a colony rough. Truncations in LPS can lead to significant attenuation in virulence and growth defects.

The interaction of TSPs with LPS are well studied in bacteriophages infecting *Salmonella enterica*. In *S. enterica*, typhoid and non-typhoid *Salmonella* are distinguished from each other. Typhoid fever is a severe infection of humans caused by *S*. Typhi or *S*. Paratyphi. These serotypes of *Salmonella enterica* are highly infectious and must not be mistaken for non-typhoid strains of *Salmonella* such as *S*. Typhimurium or *S*. Enteritidis. The latter serotypes cause Salmonellosis, a food borne infection of the gastro intestinal tract that leads to diarrhea and abdominal pain. Typhoid fever can directly spread from one patient to the other, while Salmonellosis is transmitted via contaminated food.

The TSP of bacteriophage P22, a temperate phage infecting *Salmonella*, is probably one of the best studied. P22 is a member of the *Podoviridae* family, Lederbergvirus genus and infects *Salmonella* serotypes sharing the trisaccharide repeating unit Man-Rha-Gal in the O-antigen. The TSP_P__22_ destroys the O-antigen ligand by cleaving the glycosidic bond between rhamnose and galactose and is therefore an endorhamnosidase. Crystallization of the protein revealed that the epitope binding site is much larger than that of an antibody, explaining why the affinity of the protein to the O-antigen is much higher compared to antibodies. The binding site is located in the central part of the beta-helix. The binding cleft accommodates all eight carbohydrate residues of two repeating units, reflecting an extensive contact surface. The active site of the enzyme was reported to be situated apart from the binding site (Steinbacher et al., 1996). Mutations of TSP_P__22_ at amino acid position 331 (V331G, V331A) strongly affected O-antigen binding, while mutations at position 334 (A334V, A334I) affected O-antigen binding only slightly (Baxa et al., 1999). It was suggested that the destruction of the O-antigen is useful in order to facilitate detachment of progeny virions after the infection cycle (Steinbacher et al., 1996).

Waseh et al. (2010) applied TSP_P__22_ to reduce *Salmonella* colonization in chicken. They found that TSP_P__22_ agglutinates *Salmonella* at 4°C but not at higher temperatures of 42°C, e.g., at the chicken’s body temperature. Oral administration of TSP_P__22_ reduced *Salmonella* colonization in the chicken’s gut and bacterial penetration into internal organs. Since motility seems to be implicated in colonization of host cells by bacteria, the authors analyzed the impact of TSP_P__22_ on *Salmonella* motility. They found that spread of the bacteria was indeed significantly reduced, if TSP_P__22_ was applied in soft agar plates incubated at 37°C (Waseh et al., 2010).

A very similar TSP was identified in the two bacteriophages Sf6 and HK620 (Chua et al., 1999; Freiberg et al., 2003; Barbirz et al., 2008). Both belong to the Lederbergvirus genus. Sf6 infects *Shigella flexneri*. The protein, again an endorhamnosidase, cleaves the tetrasaccharide repeat unit of the O-antigen in the Y serotype (Chua et al., 1999). In HK620, a coliphage, the TSP exhibits a highly similar secondary and tertiary structure compared to TSP_P__22_ and TSP_Sf__6_, however at the C-terminus the amino acid sequence lacks homology. In fact, TSP_HK__620_ is an endo-N-acetylglucosaminidase, which specifically cleaves the *E. coli* O18 antigen (Barbirz et al., 2008). All three proteins exhibit a conserved pg17_T__7_-like domain at the N-terminus, which connects the protein to the virion. Barbirz et al. (2008) reported a flexible linker between the N- and C-terminal domains in TSP_HK__620_. In addition, they found that the substrate-binding site of TSP_HK__620_ and TSP_P__22_ is on the face of the beta-helix, e.g., *intra*subunit, while in TSP_Sf__6_ it is located between two helices, e.g., *inter*subunit (Barbirz et al., 2008; Leiman and Molineux, 2008). It is worth noting that substrate binding in highly conserved TSPs can be achieved in different manners.

Phage SP6, a Zindervirus, is phylogenetically closely related to bacteriophages K1E, and K1-5 (Scholl et al., 2002). However, SP6 predominantly infects *Salmonella*, while K1E and K1-5 are K-type specific coliphages (Leiman et al., 2007). The TSPs of SP6, however, do not recognize capsular antigens, but bind specifically to *Salmonella* LPS (Scholl et al., 2002). Like all members of the Zinderviruses, SP6 exhibits two TSPs, Gp46 and Gp47 (Gebhart et al., 2017; Tu et al., 2017). Both are connected to the baseplate via an adaptor protein, Gp37. Gp46 is an endorhamnosidase, which enables SP6 to infect *S.* Typhimurium and *S.* Enteritidis serotypes (Majkowska-Skrobek et al., 2016). The protein is functionally similar to TSP_HK__620_, TSP_P__22_, and TSP_Sf__6_ and therefore shares a conserved C-terminus with these proteins, but it lacks the N-terminal gp17_T__7_-like domain, which connects TSP_HK__620_, TSP_P__22_, and TSP_Sf__6_ to the baseplate (Figure 2).

The second TSP in SP6, Gp47 facilitates binding of the phage to *S.* Newport and *S.* Kentucky (Gebhart et al., 2017). The Gp46, Gp47, and Gp37 complex has a V-shaped structure. Only one TSP at a time binds to the host cell surface during adsorption. Hence, if *S*. Typhimurium is replaced by *S*. Newport, the entire V-shaped complex rotates in order to move the second TSP in a downward facing position. Thereby, the Gp46 tail spike undergoes a 60° upward rotation during adsorption, while the Gp47 tail spike undergoes a 60° downward rotation. Interestingly, SP6 does also infect rough *Salmonella.* This could be due to a general low affinity of the tail spikes for the core LPS further aiding adsorption (Tu et al., 2017). A highly similar tail spike architecture was described in bacteriophages K1E and K1-5 (Leiman et al., 2007).

*Pseudomonas aeruginosa* is the host bacterium of bacteriophage LKA1, a member of the *Autographivirinae*, Phikmvvirus genus. In *P. aeruginosa* two different types of LPS are produced. The A-band and the B-band. The A-band LPS contains a conserved O polysaccharide region composed of D-rhamnose (homopolysaccharide), while the B-band O-antigen (heteropolysaccharide) structure varies among the 20 O-serotypes of *P. aeruginosa* (Rocchetta et al., 1999). LKA1 is an O5-specific bacteriophage, which exhibits an O-specific polysaccharide lyase (Gp49). This lyase specifically binds and cleaves the B-band LPS (Olszak et al., 2017). Crystal structure of the TSP Gp49 suggests that the putative substrate binding and processing site is located on the face of the beta helix. The enzyme exhibits three domains. A typical N-terminal beta-helix, which binds and cleaves the substrate, a six-stranded beta-barrel insertion domain, which deepens the substrate binding groove and participates in the creation of the active site, and a C-terminal beta-sandwich, which could also be involved in substrate binding (Olszak et al., 2017). The Gp49 is stable at temperatures of 80°C and a 1-hour incubation at pH 6.0 or 12.0 reduced its activity by 50%.

The TSP Gp49 was further tested in virulence assays using the wax moth *Galleria mellonella* as a model system. Application of the TSP reduced virulence *in vivo* and the authors demonstrated enhanced sensitivity of treated bacteria to serum killing. Moreover, the activity of the antibiotics ciprofloxacin and gentamycin, was not affected. Finally, in contrast to Dpo_ϕ15_, which specifically cleaves alginate (Cornelissen et al., 2011; Cornelissen et al., 2012), this enzyme caused biofilm degradation in the tested Pseudomonads. Hence, the authors concluded that LPS plays an important role for biofilm formation in *P. aeruginosa* (Olszak et al., 2017).

The *Vibrio cholerae* O139 infecting bacteriophage JA1 (*Podoviridae*) harbors a lyase which cleaves CPS between the GlcNAc and GalA residues. The authors discuss that the lyase may be useful for the generation of oligosaccharides, which could be effectively applied as CPS based vaccination. Since the CPS of *V. cholerae* is sensitive to acid, chemical treatment of CPS molecules could destruct epitopes that may be important to elicit protective immunity. The lyase in contrast to chemical treatment leaves these epitopes intact (Linnerborg et al., 2001).

The structure and function of the receptor-binding complex of bacteriophage CBA120, a member of the *Ackermannviridae* family, *Cvivirinae* subfamily, Kuttervirus genus was recently determined. CBA120 is an *E. coli* O157 infecting phage (Plattner et al., 2019). The production of Shiga Toxins (STX) makes infections with this particular type of *E. coli*, e.g., the Shiga Toxin producing *E. coli* (STEC), very severe. Released STX can cause kidney failure in infected patients. The disease, also referred to as hemolytic uremic syndrome (HUS), is treated with selected antibiotics, which do not enhance burst release of the toxin from the bacteria. If no antibiotic can be applied, only the symptoms can be treated, but not the cause of infection. Importantly, the production of STX is not restricted to O157, since many other O-serotypes of the bacteria, e.g., O26, O103, O104, O111, O145, and many more produce STX (Mir and Kudva, 2019).

In phage CBA120 four different TSPs, TSP1–TSP4, have been identified. TSP2 specifically binds and cleaves the O157 polysaccharide. Substrate-binding is performed in a cavity on the interface of two adjacent polypeptide chains. The enzyme then cleaves the O157 polysaccharide into Glc-GalNAc-Rha4NAc-Fuc tetrasaccharide subunits. Substrates of TSP1, TSP2, and TSP3 have been identified by bioinformatic analyses. It seems that prophage TSP sequences correlate with the O-antigen of the respective host bacterium. The analyses suggested that TSP1 might target *Salmonella enterica* serotype Minnesota or *Citrobacter freundii*. TSP3 and TSP4 seem to interact with *E. coli* O77 and O78, respectively. Indeed, infection of *S.* Minnesota, *E. coli* O77 and O78 by CBA120 could be demonstrated. The four TSPs are assembled in a branch-like structure that is attached to the baseplate of the phage. A very interesting finding was the identification of a Gp10_T__4_-like module in TSP2 and TSP4. Apparently, this particular module is needed to build up the complex branched architecture of the tail spikes (Plattner et al., 2019).

## Future Perspectives: Phage Engineering

The highly conserved architecture of TSPs in different phage genera prompted researchers to genetically engineer and exchange the proteins to alter host ranges of the respective phages (Ando et al., 2015; Lai et al., 2016; Gebhart et al., 2017; Yosef et al., 2017). Since TSPs are connected to the virion via the highly conserved N-terminal Gp17_T__7_-like domain an exchange of the C-terminal enzymatically active domain was a straightforward approach. The presence of a flexible linker between the two domains may further allow optimizing exchanges of the C-terminal domain (Barbirz et al., 2008). Due to the high diversity in the primary structure, an exchange of a domain can be challenging, as it may not be clear at which amino acid position two different domains can be exchanged. Fortunately, phage tail spikes are more and more studied on the structural level, which provides a solid basis for future structure-guided TSP engineering.

Ando et al. (2015) applied a synthetic biology strategy to reprogram the host range of T3 and T7, respectively. The entire phage genomes were assembled from smaller genome fragments and cloned into yeast artificial chromosomes. In Gp17 the C-terminal domain was exchanged and synthetic phages were created. As expected the host ranges of T3_T__7__(gp__17__)_ and T7_T__3__(gp__17__)_ were swapped. These findings suggest, that the C-terminal domain of Gp17 defines the host range in Teseptimaviruses. Therefore, new host ranges can be conferred onto Tesetpimavirus scaffolds by engineering tail spikes. In addition, the technology was also used to redirect *E. coli* phage scaffolds to target pathogenic *Yersinia* and *Klebsiella* bacteria (Ando et al., 2015).

In another study, T7 was engineered for transduction of non-host bacteria applying a mutant lacking the tail genes gp 11, 12, and 17 (Kiro et al., 2013; Yosef et al., 2017). The deletion was complemented *in trans*. Therefore, homologs of the tail fiber genes from 15 different phages were cloned into plasmids. The plasmids also encoded an antibiotic resistance marker together with a T7-packaging signal. A phage propagation strain was transformed with the plasmid and then infected with the phage. The cell lysate contained virions encapsulating the T7 genome and virions encapsulating the plasmid at a ratio of ca. 1:1. The virions were used to transduce new host bacteria. Transductants were selected due to the plasmid encoded antibiotic marker. With this platform suitable TSPs for adsorption to new permissive host strains can be selected. Moreover, the efficiency of adsorption can be enhanced if the plasmid is subjected to a random mutagenesis followed by up to three panning rounds on a new permissive host strain (Yosef et al., 2017).

The TSPs of SP6 were used to replace the tail fiber gene of an R-type pyocin. R-type pyocins are bacteriocins, which feature a P2-like myoviral phage tail including baseplate and tail fibers, but lack the capsid and the genetic material. The bacteriocin is produced and burst-released by an infected bacterium. After release, the bacteriocin binds to its target bacterium through its tail fiber proteins. Then the cell membrane is punctured by a sheath contraction and insertion of the core. The target cell dies after depolarization of the cell membrane. Gebhart et al. demonstrated that a *Pseudomonas* specific R-type pyocin, e.g., R2, can be retargeted to strains of *S*. Kentucky and *S*. Newport if Gp47_SP__6_ was used to replace the native tail fiber protein of R2, and to strains of *S*. Typhimurium and *S*. Enteritidis, if Gp46_SP__6_ was used (Gebhart et al., 2017).

Lai et al. (2016) isolated and characterized two Friunaviruses, phage AB1 and AB6, which infected different strains of the host *A. baumannii*. The viruses exhibited a high degree of conservation, except for the lyase encoding gene. The author’s exchanged ORF41_AB__1_ with ORF40_AB__6_ to generate the chimeric phage AB1tf6. As expected the host range of the chimeric phage was altered (Lai et al., 2016).

Due to the enormous progress in DNA sequencing technologies and recent developments in synthetic biology, engineering of bacteriophage genomes becomes a promising and powerful approach to optimize phage-based applications. For further reading recent review articles provide more detailed information on this matter (Chen et al., 2019a; Kilcher and Loessner, 2019).

In the context of the emerging antibiotic crises, novel approaches to tackle this crisis are urgently needed. As the structure and function of podoviral lyases is already well known, future research may explore on more complex baseplates and lyases in myo- and siphoviruses. Combining this fundamental research with the newest developments in synthetic biology, novel powerful antimicrobials such as engineered phages or optimized lyases, could be generated for future applications against life threatening bacteria.

## Author Contributions

All authors listed have made a substantial, direct and intellectual contribution to the work, and approved it for publication.

## Conflict of Interest

The authors declare that the research was conducted in the absence of any commercial or financial relationships that could be construed as a potential conflict of interest.
